# Spatial Patterns and Associations between Species Belonging to Four Genera of the Lauraceae Family

**DOI:** 10.1371/journal.pone.0111500

**Published:** 2014-11-03

**Authors:** Lin Li, Wan Hui Ye, Shi Guang Wei, Ju Yu Lian, Zhong Liang Huang

**Affiliations:** 1 Guilin University of Electronic Technology, Guilin, China; 2 South China Botanical Garden, Chinese Academy of Science, Guangzhou, China; Field Museum of Natural History, United States of America

## Abstract

Spatial distribution pattern of biological related species present unique opportunities and challenges to explain species coexistence. In this study, we explored the spatial distributions and associations among congeneric species at both the species and genus levels to explain their coexistence through examining the similarities and differences at these two levels. We first used DNA and cluster analysis to confirmed the relative relationship of eight species within a 20 ha subtropical forest in southern China. We compared Diameter at breast height (DBH) classes, aggregation intensities and spatial patterns, associations, and distributions of four closely related species pairs to reveal similarities and differences at the species and genus levels. These comparisons provided insight into the mechanisms of coexistence of these congeners. O-ring statistics were used to measure spatial patterns of species. *Ω*_0–10_, the mean conspecific density within 10 m of a tree, was used as a measure of the intensity of aggregation of a species, and g-function was used to analyze spatial associations. Our results suggested that spatial aggregations were common, but the differences between spatial patterns were reduced at the genus level. Aggregation intensity clearly reduced at the genus level. Negative association frequencies decreased at the genus level, such that independent association was commonplace among all four genera. Relationships between more closely related species appeared to be more competitive at both the species and genus levels. The importance of competition on interactions is most likely influenced by similarity in lifestyle, and the habitat diversity within the species’ distribution areas. Relatives with different lifestyles likely produce different distribution patterns through different interaction process. In order to fully understand the mechanisms generating spatial distributions of coexisting siblings, further research is required to determine the spatial patterns and associations at other classification levels.

## Introduction

The spatial distribution of species and inter-species interactions provide fundamental information for understanding species structures and coexistence in communities. One major focus of ecological research is to reveal the outcome of the interactions of biological and ecological processes by analyzing spatial distribution patterns and associations [1]–[5]. For instance, a recent study [6], which simulated spatial processes of seed dispersal and habitat association found that niche- and neutral-based interactive operations may have important roles in generating spatial patterns. Furthermore, spatial distribution of species can essentially be used to understand and model biodiversity patterns over space [7]–[13]. For example, Pofessor He and Legendre [11] and Green and Ostling [14] interpreted relationships based on the spatial patterns of individuals within their distribution and endemic-range, respectively. Most studies have utilized statistical analyses of species data to reveal processes and phenomena explaining species coexistence mechanisms. Gotelli *et al.* [15] used a general simulation model to explain the patterns and causes of species richness. The simulation approach offers new insights into the origin and maintenance of species richness patterns, combined with contemporary climate, evolutionary history and geometric constraints on global biodiversity gradients [15]. Rahbek *et al.* [16] found correlative climatic models substantially underestimate the importance of historical factors and small-scale niche-driven assembly processes in shaping contemporary species-richness patterns.

Analyses of the spatial patterns of sympatric congeneric species present unique opportunities and challenges to explain species coexistence [17]–[19]. Congeneric species, and even species within the same families stemmed from a common ancestor. Sympatric siblings usually possess many phenotypic and ecological trait similarities, and therefore, utilize analogical resources similarly. Survival under conditions of limited resources, thus requires repulsion among sibling species preventing their coexistence [20]. However, congeneric trees are commonly found to coexist within high-diversity tropical communities and low-diversity temperate communities; Inter-specific differences and ontogenetic shifts in light requirements with life-form differences may contribute to the coexistence of the Acer species in old-growth forests [21]. Partitioning of the topographic and light environments was verified may double or treble the number of species able to coexist, but no evidence that partitioning of physical habitats can explain the coexistence of these closely related species [22].

Many ecologist and evolutionary biologists devote themselves to addressing the following questions: One is what factors drive the relative distribution of congeneric trees within the same community. Competitive ability is known to influence distribution patterns of species. Species with stronger competitive ability may retain more individuals than others species, a species’ relative abundance is positively related to its competitive ability modeled by Du [23]. Also competition and facilitation can structure plant distribution and assemblages [24]. Additionally, the distributions of two strongly repulsed species within the same community usually differ. A second question is why does the abundance of coexisting sibling trees tend to differ? The literature on plant rarity often speculates that rare species are poor competitors [25], [26], resulting in their reduced abundance. Though some rare species may have low competitive ability, it should not be assumed as the cause of rarity for all rare species [27]. In fact, other studies have attributed superior competitive ability to rare species [28], [29]. The conclusions of these studies were based on a consensus that competition exists between congeneric trees and that competitive ability and action differ with spatial distribution patterns and abundance. Furthermore, habitat is most likely another important factor influencing congener distributions [30]. Large plots provide the basic element for the study of congener coexistence. A study of a 52 ha tropical rain forest plot in Borneo found habitat heterogeneity (light, established micro sites, and soil textural properties) lead to differences in tree distribution patterns, and played important roles in their species coexistence [31]. However, another 50 ha tropical plot revealed no strong evidence of a relationship between partitioning of physical habitat and 16 coexisting sibling species belonging to the family Myristicaceae [22].

Most previous research on spatial patterns and associations of related species has been performed at the species level, but biological function is influenced at multiple levels. Therefore, studies on species interactions at different levels are required. While there are little is known about species interactions at multiple levels so far. Study in tropical of Myristicaceae tree seedlings in two separate taxonomic level (species and genus) analyses were hinted that different mechanisms of coexistence among tropical tree taxa may function at different taxonomic or phylogenetic scales [32]. Here, we studied the family Lauraceae which is made up of a large number of genetically-related species. Lauraceae is also a family with a long history in East Asia. We compared the population structure, spatial patterns and associations of eight congeneric species within a large (20 ha) subtropical forest. Our objectives involved answering the following questions: (1) Do genetic relationships at the species or genus level influence the population structure of coexisting species? (2) What mechanisms control spatial distributions and associations and how do they change with scale, genetic relationship or habitat heterogeneity? (3) Do patterns in aggregation intensity response to Diameter at Breast Height (DBH) differ at the species and genus level? Finally we discuss mechanisms of coexistence among genetically related species at different relationship levels, and in different forests. This analysis will contribute to the understanding of congeneric species coexistence and diversity maintenance in subtropical forests.

## Methods

### Ethics Statement

No specific permits were required for the described field studies. The study site Dinghusan plot (DHS plot) is owned by the Chinese government and the Chinese Forest Biodiversity Monitoring Network, DHS plot is managed by South China Botanical Garden, Chinese Academy of Sciences. We can do our research works freely in these plots under the Regulations of the People’s Republic of China on Nature Reserves. Our field studies did not involve endangered or protected species.

### Study Site

The study area was located in the Dinghushan Mountain (112°30′39″–112°33′41″E, 23°09′21″–23°11′30″N) in Guangdong Province. Dinghushan was the first Nature Reserve established in China in 1956 and has been significantly important to the conservation of forest ecosystems over the past 50 years [33]. The reserve is covered by tropical-subtropical forests and is comprised of low mountains and hilly landscapes. Its total area is 1155 ha, with an altitude of 14.1–1000.3 m. Dinghushan has a south subtropical monsoon climate with a mean annual temperature of 20.9°C, and a mean monthly temperature of 12.6°C in January and 28.0°C in July. Average annual precipitation is 1929 mm, with most of the precipitation occurring between April and September. Annual evaporation is 1115 mm and relative humidity 82%.

A permanent 20 ha (400×500 m) plot called the Dinghushan plot (DHS plot) was established in the Dinghushan reserve in November 2004. Mapping of the plot mainly took place from January to March, but was completed in October 2005. Following the field protocols of the Center for Tropical Forest Science (CTFS), to identify, measure and map the trees, including all free standing trees and shrubs of at least 1 cm in DBH, we used the same methodology as in [34],The plot features rough terrain with a steep hillside in the southeast corner. Topography varies with ridge and valley in the plot and the elevation ranges from 240 to 470 meters. There were 56 families, 119 genera, 210 species and 71617 individuals counted in the first census. Thirty of the identified species were composed of solitary individuals and 110 species were made up of fewer than 20 individuals. The most abundant species *Aidia canthioides* owned 5996 individuals. Mean stand density was 3580.85 living trees and shrubs per hectare. Mean basal area was 28.24 cm^2^ per ha [33].

### Study Species

The Lauraceae family is made up of about 45 genera and 2000 to 2500 species. Almost all of these species are native to tropical and subtropical regions, with the distribution centers in Southeast Asia and Brazil. As a typical subtropical forest plot, the DHS plot has 19 species of Lauraceae belonging to 5 genera. Considering the species abundance and genetic relationship distance, eight focus species (abundance >200 each species) belonging to 4 genera, were chosen for comparisons of population structure and spatial patterns in this study. The selected species were *Cryptocarya concinna* (CRCO), *Cryptocarya chinensis* (CRCH), *Lindera metcalfiana* (LIME), *Lindera chunii* (LICH), *Machilus breviflora* (MABR), *Machilus chinensis* (MACH), *Neolitsea umbrosa* (NEUM) and *Neolitsea membranaceum* (NEME).

### Data Analysis

We used the relative neighborhood density index *Ω_r_* to characterize the distribution of eight Lauraceae species found in the plot. The *Ω_r_* is the abundance-based O-ring scaled for the species evaluated, and is calculated as 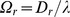 [8], *λ* is the mean density of a given species across the whole plot. Where 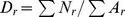, *A_r_* is the area of annulus at distance *r* and *N_r_* is the number of conspecifics within the annulus. In this study the annulus width is 10 m. Therefore, *D_r_* is the density of conspecifics as a function of distance *r*, *λ* is the mean density of a given species across the whole plot. A Monte Carlo simulation (999 iterations) was used to test the hypothesis that a distribution is not significantly different from a random distribution, i.e., *Ω_r_*  = 1. If the observed *Ω_r_* fell within the 2.5th (bottom dash-line) and 97.5th (top dash-line) quartiles, the null hypothesis was not rejected, and the species distribution within the DHS plot was concluded to be significant randomly distribution [35]. If the observed *Ω_r_* located above the top dash-line, it indicates the significant aggregation distribution. If the observed *Ω_r_* located below the bottom dash-line, it indicates the significant regular distribution.

We used DNA test results to perform cluster analysis, to quantify the genetic relationships among the species examined and accordingly group the species. DNA sequences were generated for 1–2 tagged individuals located within the DHS plot. Genomic DNA was extracted from leaf and/or bark tissue using a standard CTAB protocol [36]. We used the relative neighborhood density *Ω_r_* to assess spatial patterns and *Ω_0–10_*, the mean conspecific density within 10 m of a tree, as a measure of the intensity of aggregation of a species [8]. And then we compared spatial intensity of aggregations of trees of different DBH classes at species and genus levels. The DBH classes were binned at 2 cm intervals. Growth type, shade tolerance and life history traits rooted in empirical data from field investigation personnel with long-term experience.

Finally we used bivariate pair-correlation function (*g_12_*(*r*)) to analyze spatial associations among and between species (within the four genera). The bivariate pair-correlation function *g_12_*(*r*) is the analogy to Ripley’s *K*_12_(*r*) [37], but it replaced the circles of radius *r* by rings with radius *r*. The function *g*_12_(*r*) quantifies the type of spatial association between species 1 species 2:
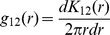



For a bivariate pattern of two objective species, *g*_12_(*r*) = 1 indicates non-association (independence), at distance r, and *g*_12_(*r*)>1 indicates a positive association between the two species at given distance r, whereas *g*_12_(*r*)<1 indicates a negative spatial interaction (spatial repulsion or segregation) between the two species at distances *r* [38]. Here the annulus width r is 10 meters.

## Results

### Population Structure

The abundance of the eight study species ranged from 223 individuals of NEME to 4478 of CRCO (Table 1). Total numbers of individuals were 13362. Two *Cryptocarya* species were much more abundant than the *Lindera*, *Machilus* and *Neolitsea* species. The total abundance of *Cryptocarya* was also much greater than for the other three genera. The basal areas of CRCH and MACH were much larger than for the other species.

**Table 1:** Population structure of the eight Lauraceae species studied in the DHS plot.

Species name	Sp. code	Genera	Growth type	Ind.	Base area (m^2^/ha)	Base area/ind	Shade tolerance order
*Cryptocarya concinna*	CRCO	*Cryptocarya*	Understory	4478	1703.21	0.38	2
*Cryptocarya chinensis*	CRCH	*Cryptocarya*	Upperstory	2557	11238.98	4.40	1
*Lindera metcalfiana*	LIME	*Lindera*	Midstory	2118	1789.99	0.85	7
*Lindera chunii*	LICH	*Lindera*	Midstory	1302	1092.76	0.84	6
*Machilus breviflora*	MABR	*Machilus*	Upperstory	800	3261.17	4.08	4
*Machilus chinensis*	MACH	*Machilus*	Upperstory	532	8250.27	15.51	3
*Neolitsea umbrosa*	NEUM	*Neolitsea*	Understory	1352	163.85	0.12	8
*Neolitsea membranaceum*	NEME	*Neolitsea*	Understory	223	125.53	0.56	5

**Figure 1:**
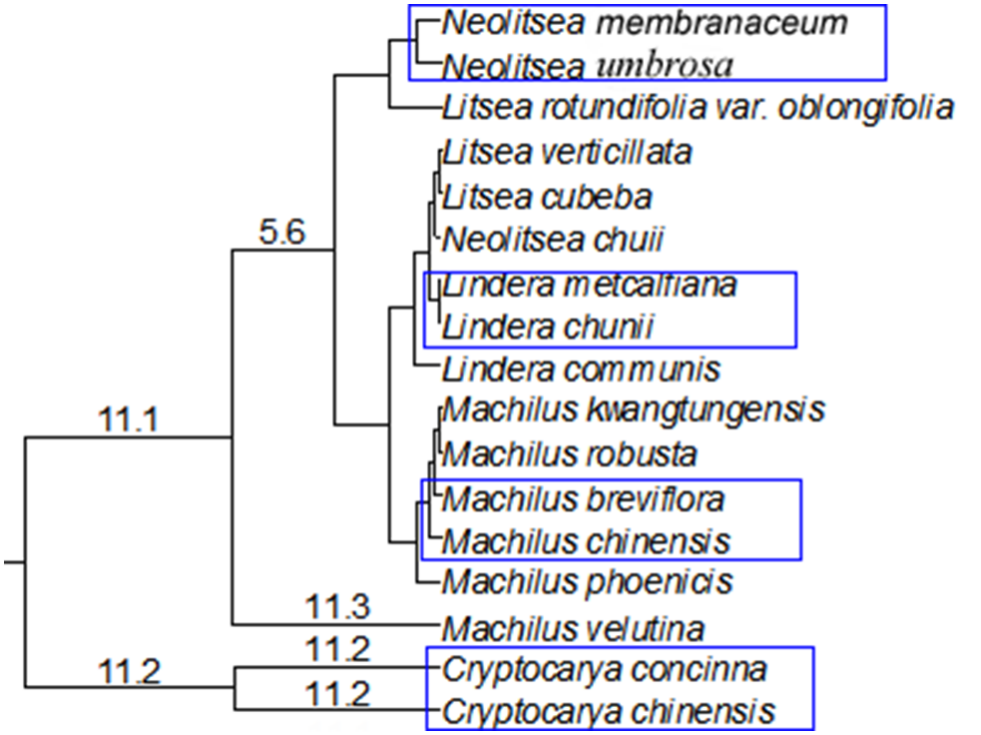
Genetic relationships of Lauraceae species. The numbers related clustering coefficient. The eight species enclosed within rectangles are the same species in Table 1.

Cluster analysis for DNA test results showed the closest relative of each of the eight species was within the same genus (Fig. 1). However, the degree of within genus species relatedness differed among the four genera. Relatedness was greatest for *Lindera* and *Machilus*, then *Neolitsea*, followed by *Cryptocarya*.

**Figure 2:**
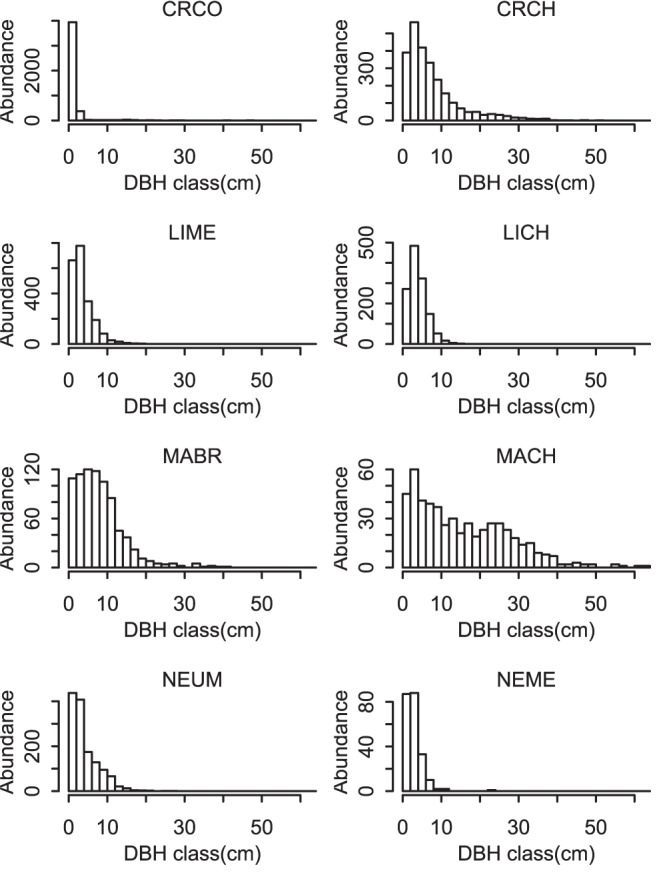
Size (DBH) class of the eight Lauraceae species belonging to four genera.

**Figure 3:**
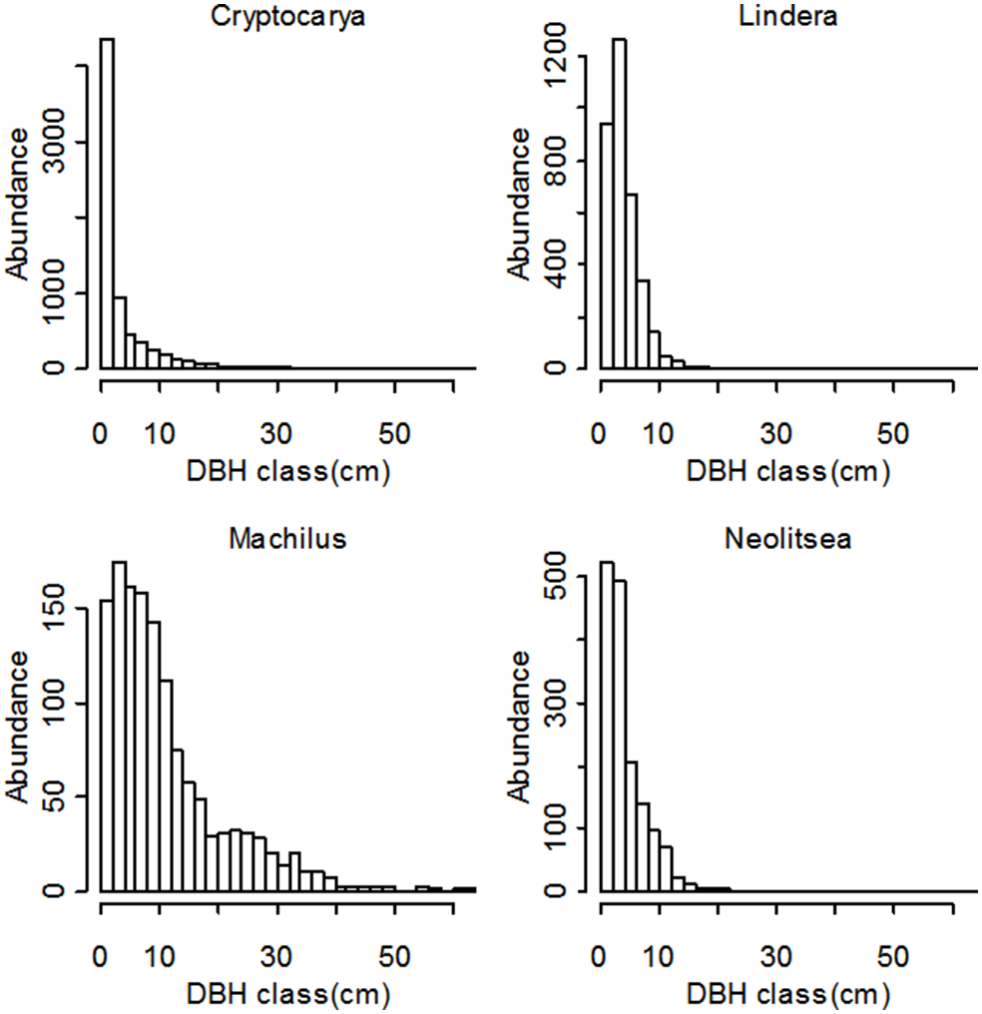
Size (DBH) class of the four genera including species from Table 1.

At the species level, eight species showed different size class distributions (Fig. 2). Using a DBH of 2 cm to compare individual’s structure of congeneric species, we found CRCO and CRCH were distinctly different. Individuals of CRCO tended to have DBH less than 4 cm and mostly were aggregated within the range of 1 to 2 cm DBH. CRCH, MABA and MACH were liked a reversed DBH J-shaped distribution. LIME, LICH, NEUM and NEME were liked a DBH L-shaped distribution. At the genera level, *Cryptocarya*, *Lindera* and *Neolitsea* had a distinct L-shaped DBH distribution (Fig. 3). *Machilus* had a larger number of big trees than the other three genera, and showed like a reversed J-shaped DBH distribution.

### Spatial Patterns

**Figure 4:**
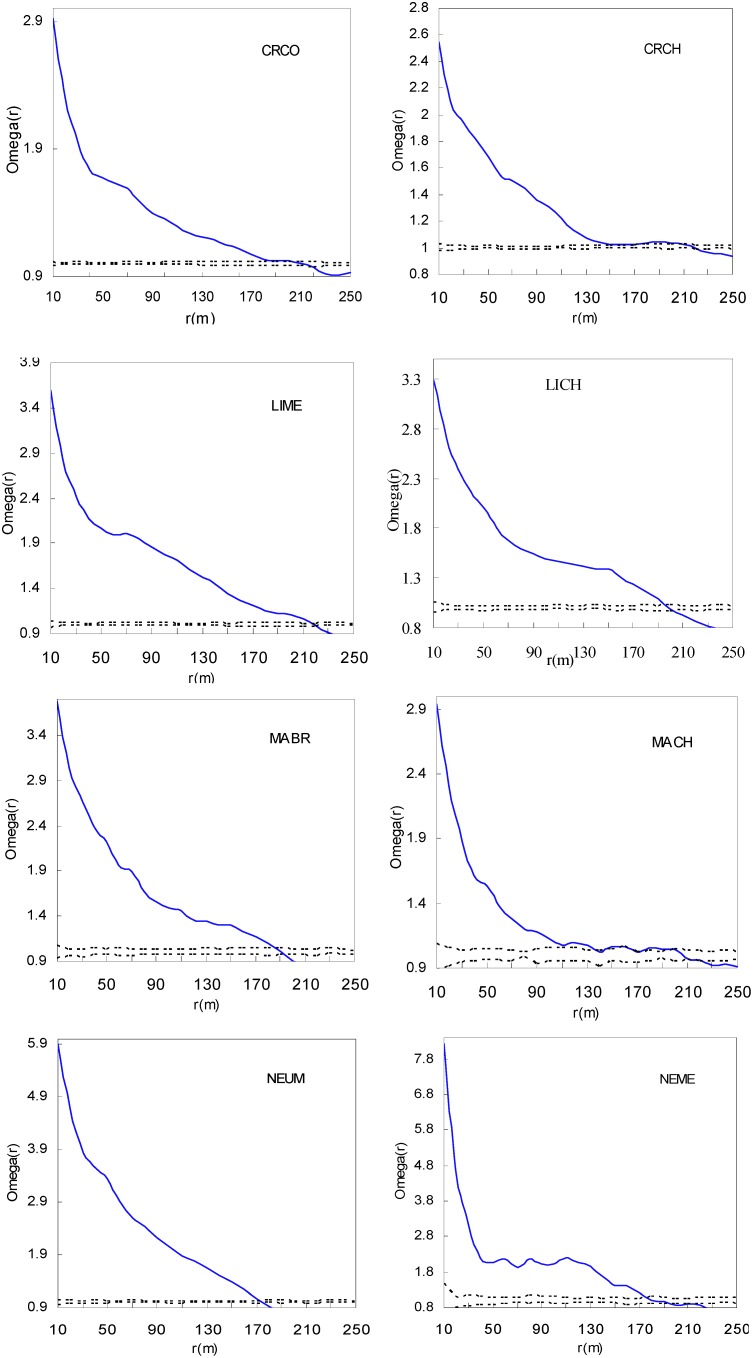
Spatial patterns of eight Lauraceae species. The solid line curve is the *Ω_r_* value. Dashed lines correspond to the confidence intervals generated from 999 Monte Carlo simulations under the null hypothesis of complete spatial randomness. See Table 1 for Species codes.

The eight study species showed aggregation patterns in the DHS plot (Fig. 4), especially at small scales. CRCO, CRCH, LIME and LICH showed patterns of aggregation at scales 0–200 m, then became random, and then regular patterns as scale increased (Fig. 4). MABR and MACH showed significant aggregated patterns at scales of 0–150 m, which then became random, then regular with increasing spatial scale. NEUM and NEME were clumped at scales less than 175 m, then NEUM became random and quickly showed regular patterns at scales >180 m. NEME, on the other hand, had a random distribution at scales of 175–200 m, then became regular as scale increased (Fig. 4).

**Figure 5:**
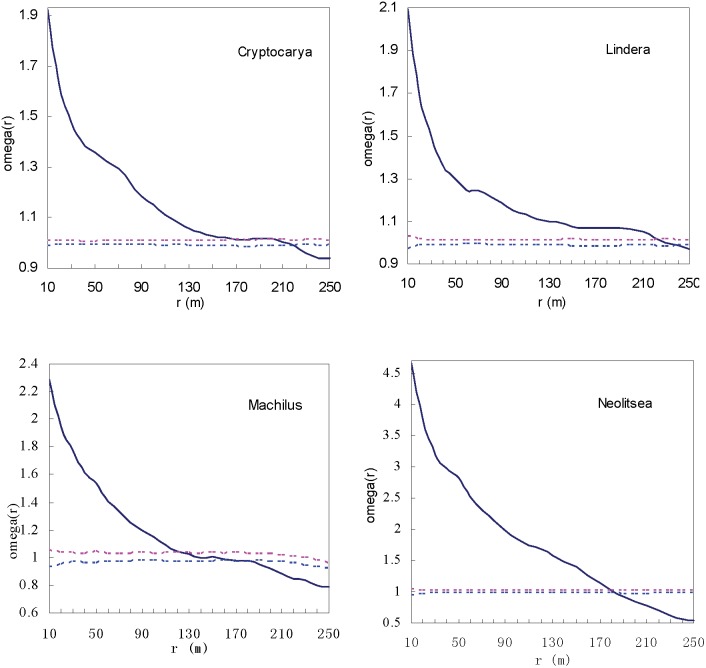
Spatial patterns of the four genera of Lauraceae species. The solid line curve is the *Ω*_r_ value. Dashed lines correspond to the confidence intervals generated from 999 Monte Carlo simulations under the null hypothesis of complete spatial randomness.

When examined at the genera level, the differences in spatial patterns were reduced. The four genera showed similar tendencies (Fig. 5). Aggregation patterns were at small and middle scales (*Cryptocarya*<200 m, *Lindera*<220 m, *Machilus*<160 m and *Neolitsea*<180 m), then random and then regular at larger scales, corresponding to their spatial pattern in the plot (Fig. 5).

**Figure 6:**
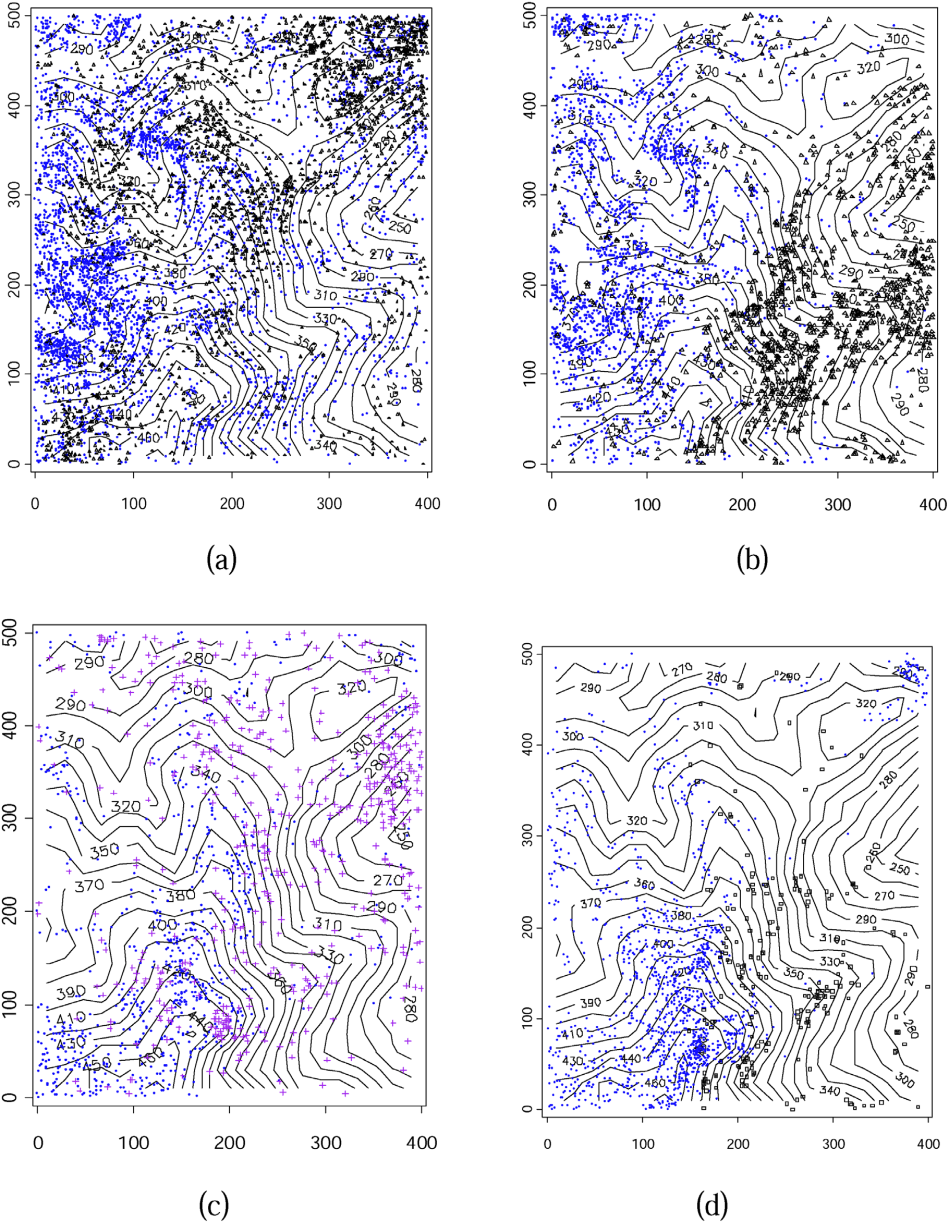
Spatial distribution of the eight species belonging to four genera in relation to the topography of the DHS plot. (a) *Cryptocarya*, dots represent CRCO and triangles represent CRCH; (b) *Lindera*, dots represent LIME and triangles represent LICH; (c) *Machilus*, circles represent MABR, crosses represent MACH. (d) *Neolitsea*, dots represent NEUM and triangles represent NEME.

Each species’ spatial distribution corresponded with its spatial patterns within the plot (Fig. 6). CRCO and CRCH were relatively widely distributed throughout entire plot, and, though their distributions did not overlap at small spatial scale, the distribution of each complemented the other at middle to large spatial scales. LIME was located mainly in the western part of the plot, whereas LICH was in the eastern part, and thus spatially not overlapping. MABR and MACH were mosaicly distributed at small to middle spatial scales. MABR was abundant at the top of mountain, located in the southwest corner of plot, and small numbers of MACH existed in complement space with MABR. NEUM was concentrated in the southwest whereas NEME was in the southeast corner, and thus the distribution of both was nearly distinct. *Cryptocarya* and *Lindera* were distributed widely throughout the plot, whereas *Machilus* and *Neolitsea* were relatively concentrated in the southern part of the plot, *Neolitsea* was especially rare in the northern part of the plot.

### Aggregation Intensity at Species and Genera Levels

The aggregation intensity as measured by *Ω*_0–10_ clearly decreased from the species to the genus level (Table 2). For example, *Cryptocarya* (*Ω*_0–10_ = 1.93) was lower than that of species CRCO (*Ω*_0–10_ = 2.91) and CRCH (*Ω*_0–10_ = 2.55). The other three genera displayed similar trends. *Cryptocarya* had the lowest *Ω*_0–10_ and *Neolitsea* (*Ω*_0–10_ = 4.67) had the highest *Ω*_0–10_ of the four genera. Species of all four genera showed tendencies of congeneric species aggregations within parts of the plot. Though the locations of the aggregates differed with species, individuals from different species inside congeners were fewer overlapped distribution (Fig. 6).

**Table 2:** Statistic *Ω*_0–10_ of eight Lauraceae species at the species and genus level (Mixed congeneric species individuals).

Genera	*Ω*_0–10_	Spcode	*Ω*_0–10_
*Cryptocarya*	1.93	CRCO	2.91
		CRCH	2.55
*Lindera*	2.09	LIME	3.59
		LICH	3.29
*Machilus*	2.29	MABR	3.78
		MACH	2.96
*Neolitsea*	4.67	NEUM	5.93
		NEME	8.29

### Relationship between Aggregation Intensity and DBH at the Genera Level

**Figure 7:**
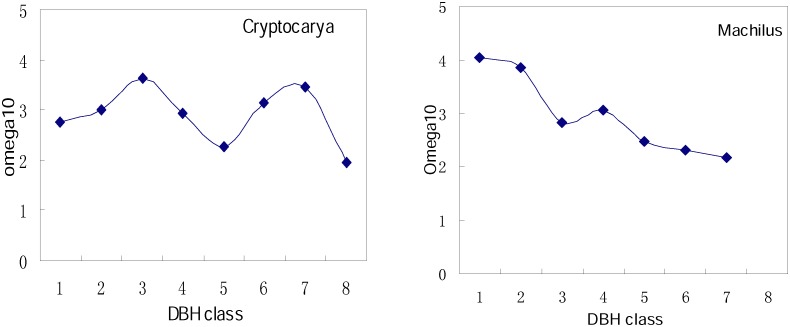
Relationships between *Ω*_0–10_ and DBH for *Cryptocarya* and *Machilus*. DBH classes were 3 cm increment bins beginning at 1 cm.

Where more than 50 individuals made up a DBH class, *Ω*_0–10_ was examined (Fig. 7). Results showed differences in the change in *Ω*_0–10_ with DBH group between *Cryptocarya* and *Machilus*. Although aggregation intensity of these two genera similarly tended to decrease from small to large DBH, the curves differed. *Cryptocarya* showed a wavelike decrease, compared to *Machilus*, appeared to linearly decrease with increasing DBH.

### Spatial Associations

**Figure 8:**
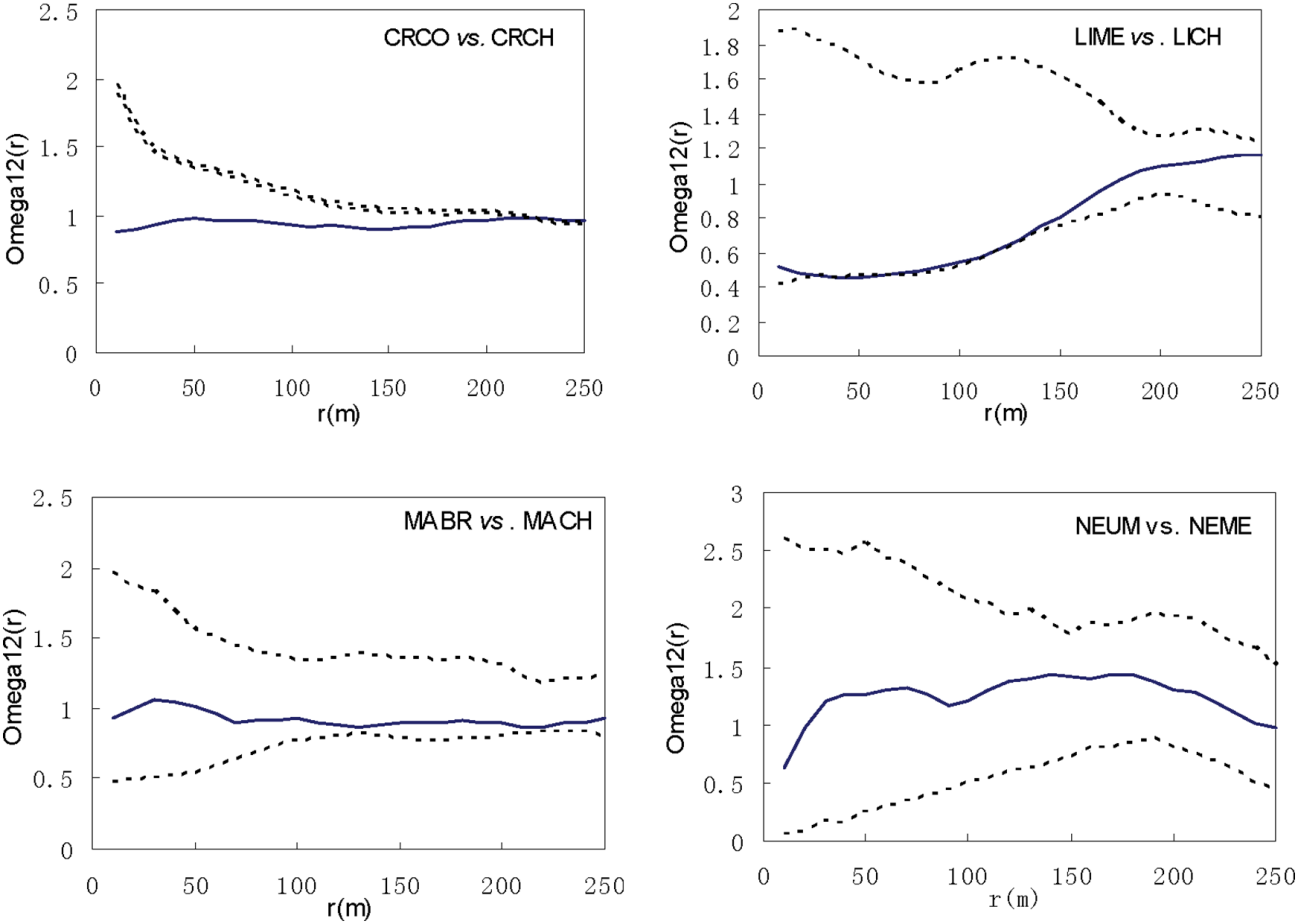
Spatial associations between Lauraceae species within genera. Solid line curve represents the *Ω_r_* value. Dashed lines correspond to the confidence intervals generated from 999 Monte Carlo simulations under the null hypothesis of complete spatial randomness.

Spatial associations of the four pairs of closely relative species showed no positive associations (Fig. 8). CRCO and CRCH were exceptionally negatively associated until scales greater than 210 m. LIME and LICH showed a negative association at scales of 20–140 m. The other two pairs were independently distributed within the plot at every spatial scale.

**Figure 9:**
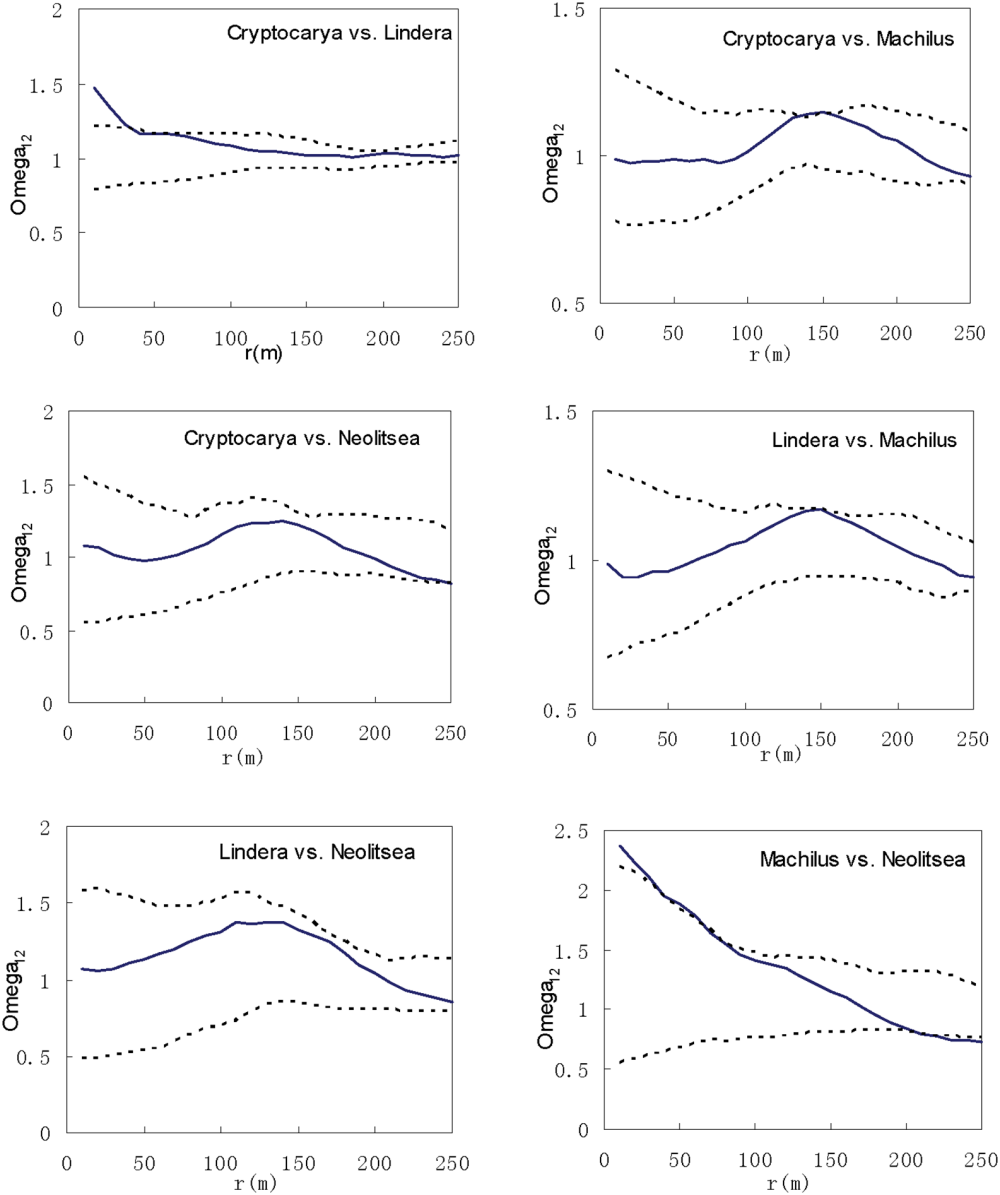
Spatial associations among genera of Lauraceae species. The Solid line curve represents the *Ω_r_* value. Dashed lines correspond to the confidence intervals generated from 999 Monte Carlo simulations under the null hypothesis of complete spatial randomness.

Independent association was commonplace among four genera (Fig. 9). Genera pairs were showed independent association, except for *Cryptocarya* and *Lindera* were positively distributed at scale <40 m, and *Machilus* and *Neolitsea* were showed positively association at scale <60 m.

## Discussion

The spatial distribution patterns of closely related congeneric trees in subtropical forests have important implications for how species interact with each other and their performance at different genetic levels. Closely related congeneric species originate from a common ancestor and, because they share many similar phenotypic and ecological traits, they tend to utilize a similar set of resources in similar ways. Therefore, they may exhibit relatively intense interspecific competition that limits their coexistence [23], [29]. The spatial distributions of and associations between the four closely relative pairs studied here suggested that closer related species would repel each other when resources are limited. The degree of competition between species pairs, would be dependent on the similarities in their lifestyles [15], and also be subject to the diversity of habitat and small-scale niche-driven assembly processes in shaping contemporary species-richness patterns within their distribution area [16]. Relatives with different lifestyles would produce different distribution patterns through different interaction processes**.** CRCO and CRCH, and LIME and LICH were showed a negative association at most scales within the sampled plot (Fig. 8). These two closely relative pairs showed inter-specific competition in that they had different spatial distribution patterns (Fig. 6).

Long term natural selection may have driven relative trees to develop different, but mutually beneficial lifestyles resulting in mutual attraction. Congeneric trees are commonly found to coexist within different communities [21], [22]. There is evidence that congeneric species can coexist if traits have diverged within the genus. When traits have diverged within the genus, the niche overlap is reduced and competition relaxed, thus allowing the coexistence of congeneric species [39]. Also, genetic differences at the subspecies level might lead to genetic subgroups taking advantage of environmental specialization, affecting spatial distribution above the species level [40]. Habitat is most likely another important factor influencing congener distributions [30]. Light, established micro sites, and soil textural properties played important roles in tree distribution patterns, and in their coexistence [31]. Habitat specialization plays an important role in maintaining the diversity of this species-rich subtropical forest. Former study found 83% of the species were related to topographic variables [41].

Inter-specific differences and ontogenetic shifts led to the law of spatial patterns and associations changed from genus level to species level. Aggregated distributions of species is a widespread pattern in nature, observed in both tropical plant communities with diverse species and temperate plant communities with relatively few species [10], [42], [43], [44]. Four genera provided further evidence of clumped patterns both at species and genera levels in a subtropical forest. Eight species showed significant aggregation patterns, especially at small scales (Fig. 4 and Fig. 6). At the genus level, differences in spatial patterns were reduced, and significant aggregation patterns existed at small and middle spatial scales (Fig. 5). The aggregation intensity varied among species (or genera), and reduced at the genus level. Furthermore, aggregation intensity of congeneric individuals was much lower than for species within genera. Though the four congeneric species pairs displayed different biotic or abiotic processes to enhance or attenuate their drive to repulse each other, then formed current pattern. Repulsive interactions among relatives were seen through decreased aggregation intensity at the genera level (Table 2).

The mechanisms behind species coexistence has been explored at the species level and rarely above or below species level. Indeed the genetic influences at the species level produced a qualitative change in species distribution. However, the genetic influence was not restricted to the species level. From individual, to species, to genera and even to higher levels, genetic influences and variation from natural selection must affect species coexistence. Results differed at different levels. Therefore, information from more levels is required to understand species coexistence mechanisms. For example, a sub-species level analysis found genetic subgroups of *Castanopsis chinensis* that showed environmental specialization; a species level analysis would not have detected this association [40].

To our knowledge, the present study is the first to trace interactions between and distributions of congeneric species at both the species and genus levels, with the aim of explaining their coexistence through examining the similarities and difference at these two levels. In our study, this was specifically achieved through comparisons of DBH classes, aggregation intensities and spatial patterns, associations and distributions of eight Lauraceae species at both the species and genus levels. DBH structure was more stable at the genus than species level. Individuals at both levels were aggregated, while aggregation intensity at the genus level was lower than for species within genera. Previous studies have found aggregation intensity to clearly decrease with DBH in DHS plot [35]. Here we also found aggregation intensity at the genus level tended to decrease from small to large DBH. Spatial associations within genera showed no positive associations, with two pairs (CRCO and CRCH, LIME and LICH) possessing negative associations. Associations among genera were almost never negative, often independent, but sometimes positive (e.g., *Cryptocarya* and *Lindera* and *Machilus* and *Neolitsea*) at the same spatial scales. As the level of examination increases from species to genus level, the negative associations become less common due to increasingly distant relationships between the compared groups.

Former studies of congeneric species interactions focused on testing and verifying whether or not interspecific competition existed. The conditions, such as resource availability and number of potential competitors, required to produce competitive relationship have been neglected up to now. Perhaps competition appeared more frequently in high than low diversity communities because a large number competitors for single resource units lead to significant competition. Studies in tropical forest communities showed interspecific competition should be much stronger among congeneric species, especially at small scales [20], [45]. In subtropical forest no positive spatial associations within genera and two pairs of negative associations were found. In the central Aegean archipelago strong evidence for widespread competition among congeneric species were not found, and most communities investigated show no significant patterns of species associations [46]. Temperate plant communities with few species showed slightly negative associations at small scales, indicating interspecific competition, few species pairs showed positive associations [47]. There maybe sufficient resources for the coexistence of congeneric species in low diversity communities with relatively homogeneous environmental conditions. Among general congeneric species pairs no negative associations occurred, and even some positive correlations were reported [47].

## Conclusion

From the study of eight Lauraceae species at genus and species level, we compared DBH classes, aggregation intensities and spatial patterns, associations and distributions of four pairs of these related species at both the species and genus level, to reveal similarities and differences at the two levels of relatedness. In conclusion, spatial aggregations were common, and the differences in spatial patterns were reduced at genus, relative to species level. The aggregation intensity clearly reduced at the genus level, the *Ω*_0–10_ value of all four genera was lower than the *Ω*_0–10_ value of the species within each genera. Aggregation intensity decreased with increasing DBH both at the species and genus levels, but due to the differences in DBH structure and growth type, the pattern of aggregation intensity change with DBH differed between individuals of two genera. Spatial associations between the four pairs of closely relative species showed no positive associations, and two pairs showed significant negative associations at several scales. The frequency of negative associations decreased at genus relative to species level, and independent associations were common among the four genera. Negative association illustrated competitive effect were existed in these negatively correlated relative species, however, coexistence mechanism of relative species at different levels was really a very complicated question. It’s a good start, in order to fully understand the mechanisms generating distribution patterns of coexisting sibling trees, further investigation into interactions between coexisting relatives at scales ranging from subspecies to families is required, and even in combination with ecological and life-history data and experimental data.
